# Unveiling Novel RecO Distant Orthologues Involved in Homologous Recombination

**DOI:** 10.1371/journal.pgen.1000146

**Published:** 2008-08-01

**Authors:** Stéphanie Marsin, Aurélie Mathieu, Thierry Kortulewski, Raphaël Guérois, J. Pablo Radicella

**Affiliations:** 1 CEA, Institut de Radiobiologie Cellulaire et Moléculaire, UMR217 CNRS/CEA, Fontenay aux Roses, France; 2 CEA, iBiTecS, URA 2096, SB2SM, Laboratoire de Biologie Structurale et Radiobiologie, Gif sur Yvette, France; Université Paris Descartes, INSERM U571, France

## Abstract

The generation of a RecA filament on single-stranded DNA is a critical step in homologous recombination. Two main pathways leading to the formation of the nucleofilament have been identified in bacteria, based on the protein complexes mediating RecA loading: RecBCD (AddAB) and RecFOR. Many bacterial species seem to lack some of the components involved in these complexes. The current annotation of the *Helicobacter pylori* genome suggests that this highly diverse bacterial pathogen has a reduced set of recombination mediator proteins. While it is now clear that homologous recombination plays a critical role in generating *H. pylori* diversity by allowing genomic DNA rearrangements and integration through transformation of exogenous DNA into the chromosome, no complete mediator complex is deduced from the sequence of its genome. Here we show by bioinformatics analysis the presence of a RecO remote orthologue that allowed the identification of a new set of RecO proteins present in all bacterial species where a RecR but not RecO was previously identified. HpRecO shares less than 15% identity with previously characterized homologues. Genetic dissection of recombination pathways shows that this novel RecO and the remote RecB homologue present in *H. pylori* are functional in repair and in RecA-dependent intrachromosomal recombination, defining two initiation pathways with little overlap. We found, however, that neither RecOR nor RecB contributes to transformation, suggesting the presence of a third, specialized, RecA-dependent pathway responsible for the integration of transforming DNA into the chromosome of this naturally competent bacteria. These results provide insight into the mechanisms that this successful pathogen uses to generate genetic diversity and adapt to changing environments and new hosts.

## Author Summary

Homologous recombination plays an essential role in maintaining genomic integrity and in allowing genetic diversity within a population. In the case of bacteria, two main pathways for the initiation of recombination have been described. These pathways are defined by the protein complexes, present in the model systems *Escherichia coli* and *Bacillus subtilis*, that allow the loading of RecA onto single-stranded DNA to engage it in the annealing process. Depending on the DNA substrate, either RecFOR or RecBCD (AddAB) act as mediators for RecA filament formation. However, analysis of the completed bacterial genome sequences shows that in many species, not all of the components seem to be present. One such example is the human pathogen *Helicobacter pylori*, where homologous recombination is known to play a critical role in its high genetic diversity by facilitating intrachromosomal rearrangements and allowing integration of exogenous DNA into its genome. In this species, classical sequence comparisons have only identified RecR and RecB mediator genes. Here we show that *H. pylori* possesses a RecO functional orthologue. This finding allowed the identification of remote homologues of RecO in all those bacterial species where RecR but no RecO was previously found.

## Introduction

Homologous recombination (HR) is an essential mechanism for both, maintenance of genetic information and genome evolution. HR allows cells to maintain its genetic integrity in response to DNA damage. HR also plays a critical role in the generation of genetic variants, allowing evolution and adaptation. This is particularly true in prokaryotes where genomic diversity creates a phenotypically diverse population from which the most fit variants are selected. In the case of pathogens, these mechanisms allow adaptation of bacteria to changing environments and new hosts therefore contributing to their virulence and pathogenicity.

The obligate human pathogen *Helicobacter pylori* colonizes the stomach of about half the human population, resulting in chronic gastritis, leading in some patients to peptic ulcers and, in a small fraction of cases, to cancer. The rapid adaptation of *H. pylori* to the changing gastric environment within a host or to new hosts, suggests a particularly enhanced ability to change. Indeed, *H. pylori* is one of the most genetically diverse bacterial species. At the origin of such diversity are both elevated mutation rates and high recombination frequencies [1],[2]. Incorporation of DNA sequences by HR into the *H. pylori* chromosome is crucial for horizontal gene transfer between strains colonizing the same host [3]. This process, facilitated by the species natural competence, is believed to be the cause of its panmitic population structure [4],[5]. Analysis of the genomic sequences has also underlined the importance of intragenomic chromosomal rearrangements mediated by HR [6]–[8].

HR basic enzymatic steps are conserved in all organisms. Most of our knowledge on the molecular mechanisms underlying HR in bacteria comes from the two model systems, *Escherichia coli* [9] and the gram-positive competent bacterium, *Bacillus subtilis* [10]. The central step in recombination (synapsis) is the DNA strand exchange mediated by the nucleoprotein filament formed by RecA on single-stranded DNA [11]. In *H. pylori*, the *recA* gene was identified and shown to be involved in repair of UV-damaged DNA and incorporation of DNA during transformation [12],[13]. Although it shares 58% identity to *E. coli recA*, the *H. pylori* protein needs to be modified post-translationally to be fully active [14]. The product of strand exchange is a branched DNA molecule named Holliday junction that is processed by the RuvAB helicase and the RuvC resolvase. The RuvABC complex is also present in *H. pylori*, where a *ruvC* mutant displays increased sensitivity to DNA-damaging agents [15] and is defective in colonization, indicating the importance of HR for infection [16].

While RecA and the Holliday junction resolvases are nearly ubiquitous in bacteria, this is not the case for proteins involved in presynaptic steps that lead to a RecA filament on single-stranded DNA [17]. In *E. coli*, two major DNA recombination pathways coexist and are complementary, the RecFOR and the RecBCD pathways. Although starting from distinct substrates, in both cases the participating proteins act to facilitate RecA nucleation on ssDNA [18]. The RecBCD pathway is needed for the repair of double strand breaks and to process regressed forks. Consistently, *E. coli* mutants with null mutations in *recB* or *recC* genes have reduced viability (around 30%) and resistance to DNA-damaging agents such as ionizing radiation. *recBC* mutants are also deficient in homologous recombination following conjugation or transduction whereas *recD* mutations display a hyper-recombination phenotype in these assays [9]. AddAB is the functional analog of RecBCD complex in *B. subtilis* [10]. The *add* mutants are less sensitive to UV radiation compared to *E. coli recBC* mutants and recombination during transformation is almost unaffected [11]. The RecFOR pathway is essential for post-replication repair of gaps and restart of replication following UV damage. In *E. coli*, genetic analysis of UV and gamma irradiation resistance puts *recF*, *recO*, and *recR* genes into the same epistasis group. However, neither *recF*, *recO* nor *recR* mutants shows a decrease in HR during conjugation or transduction [9],[19],[20]. In *B. subtilis*, where the three genes are also present, the mutants are much more sensitive to UV when compared to the *E. coli* mutants [11].

There is no experimental data on the HR initiation steps in *H. pylori*. Remarkably, upon inspection of the three sequenced strains, key proteins of the two initiation pathways appear to be missing [21]–[23]. The RecBCD complex would only be represented by a protein (HP1553 in strain 26695) displaying poor identity to *E. coli* RecB. Not only the RecC and RecD components seem absent but, while in *E. coli recBC* mutants tend to yield suppressor mutations by activating a prophage or by inactivating nucleases, such alternative systems, represented by exonuclease I or the SbcCD nuclease, were not found by genome analysis in *H. pylori*. For the RecFOR pathway, although a RecR homologue (HP0925 in strain 26695) has been identified, neither RecO nor RecF have been detected by sequence analysis. This raises the interesting possibility that other initiation pathway(s) might exist in *H. pylori.* Alternatively, the missing activities might be harbored by extremely diverged proteins, impossible to detect with currently used alignment algorithms.

The supposed absence of a number of key HR proteins in *H. pylori*, in spite of the importance of recombination for this species, together with its reduced number of genes (less than half the number present in *E. coli)* [21],[23] make of this species an interesting model for the study of recombination mechanisms. This, added to the importance of HR for genetic diversity and adaptation capacity of *H. pylori*, prompted us to analyse the HR mediator genes present in this pathogen and their role in genetic stability maintenance and horizontal gene transfer.

## Results

### Identification of a Novel RecO Family

Despite the functional interdependency between RecO and RecR and the presence of a well conserved RecR orthologue, sequence analyses of the *H. pylori* genomes [21]–[23] failed to detect a RecO homologue. This observation extends to all the e-proteobacteria family members for which no RecO orthologues were detected among the 320 homologous sequences that could be retrieved after the five iterations of PSI-Blast required to reach convergence. The substitution rates for the RecO homologues are among the highest in the set of proteins involved in the bacterial homologous recombination systems analyzed by Rocha et al. [17]. We thus explored the possibility that a distant homologue of the *recO* gene may have been missed from traditional PSI-Blast analyses. The 35 sequences achieving an e-value above the significant threshold 10^−3^ up to an e-value of 10 were considered. At an e-value of 1.5, a sequence from *Campylobacter fetus* caught our attention because it belonged to the same e-proteobacteria group as *Helicobacter pylori*. No other possible candidate could be identified. Using the *C. fetus* sequence as a seed, we could retrieve homologous sequences from *H. pylori* with high confidence. The alignment made of only e-proteobacteria sequences was compared with the other RecO homologues sequences using the HHalign profile-profile comparison method and yielded high probability that these sequences were evolutionary related (probability 96.2 % with e-value of 5.10^−4^) [24]. The *H. pylori* gene *HP0951* initially annotated as a hypothetical protein was found as a likely *recO* orthologue, although its sequence identity with the *E. coli* protein is lower than 15 %. Such strategy for remote homologue detection was also successfully used in recent studies [25].

**Figure 1:**
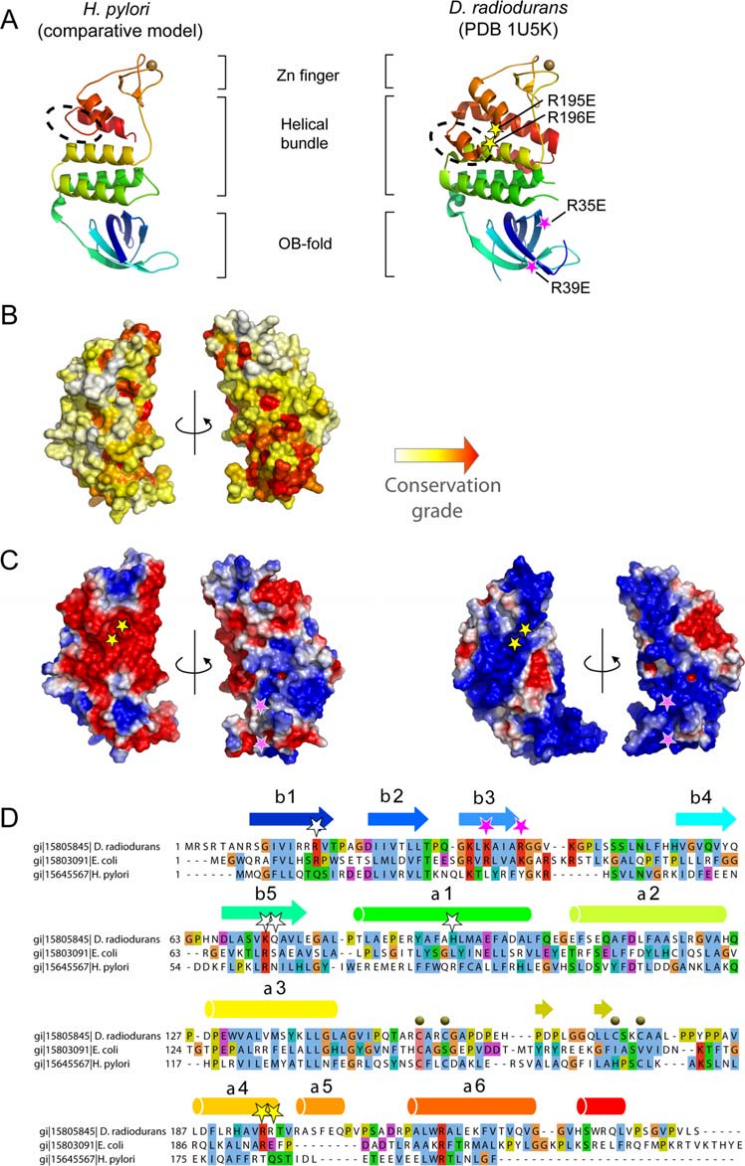
Comparison between *H. pylori* homologue and the *D. radiodurans* RecO. (A) Comparison between the experimental structure of the model of the *H. pylori* homologue and the *D. radiodurans* RecO represented as ribbon and colored from the N- to the C-terminus from blue to red, respectively. The dashed circle indicates the major structural variation between both proteins corresponding to the absence of the helix α5 in *H. pylori* homologue. Pink stars indicate the position of the two mutations that disrupted RecO DNA binding properties while yellow stars indicate the positions of two mutations that only partially affected DrRecO DNA binding [26]. (B) Evolutionary rates calculated at each position of the global RecO multiple sequence alignment mapped onto the surface of *H. pylori* model shown under two opposite orientations. Conservation grade was calculated using the rate4site algorithm [44] and increases from white to red for variable to highly conserved positions, respectively. (C) Representation of the electrostatic potential calculated with the APBS program and projected on the molecular surface of RecO and shown under two opposite orientations. (D) Subset of the multiple sequence alignment for DrRecO, EcRecO and HpRecO. Secondary structures were colored with respect to the ribbon representation in panel A.

*Deinococcus radiodurans* RecO, for which a structure is available [26],[27], is composed of three modules namely an OB-fold, a helical bundle and a Zn finger. A multiple sequence alignment of the RecO family was built and optimized manually taking into account the structural information from *D. radiodurans* RecO tri-dimensional structure. Modeling of the *H. pylori* RecO homologue predicts the conservation of all these modules, except for the helical bundle for which no equivalent of helices α5 and α7 could be found (Figure 1A, dashed circle). As a comparison, a structural model of *Escherichia coli* RecO was also built using the same comparative modeling methods (Figure S2). Neither was helix α5 detected in the *E. coli* homologue suggesting it plays a minor role in RecO function. The conservation grade represented at the surface of the *H. pylori* model (Figure 1B) highlights in red that functionally important positions cluster on one side of the OB-fold together with more scattered positions above this domain. These scattered positions correspond for instance to an invariant glycine at the C-terminus of helix 3 and to the [CHST]xx[CST] motifs involved in zinc coordination in the Zn finger domain (labeled as dark spheres in Figure 1D). Although *H. pylori* RecO is highly divergent, the glycine (G134) and the zinc chelating residues appear conserved (Figure 1D and Figure S1). In contrast, the basic residues labeled by pink stars in Figure 1A, C, and D are mutated in *H. pylori* although they were identified as key DNA binding residues in the mutational analysis of DrRecO [26],[27]. This observation suggests that the DNA binding properties may be altered in HpRecO. The comparison between the global electrostatic potentials calculated for HpRecO and DrRecO provides a complementary view (Figure 1C). DrRecO exhibits a surface globally highly positively charged. The basic character of the electrostatic potential was also observed from the *Escherichia coli* RecO model (Figure S2). Such property does not seem conserved over most of the surface of the *H. pylori* homologue since HpRecO exhibits large acidic patches (Figure 1C). However, focusing at the OB-fold, the basic character observed in both DrRecO and EcRecO is still present in HpRecO. This observation suggests that HpRecO may still bind DNA. The role of the basic residues labeled as pink stars may be delocalized at other positions such as Arg13, Arg20, Arg32 or Arg37, maintaining an overall positive electrostatic field in the same region as for the other RecO homologues.

Recent comparative and evolutionary analysis of the bacterial HR systems suggested that, besides *H. pylori*, several bacterial species conserved a RecR but not a RecO homologue [17]. A subset of the aligned sequences is presented in Figure S1. In cases where RecR was absent, like in *Mycoplasma pneumoniae*, no trace of RecO could be found. Existence of putative remote homologues for RecR that may explain its absence can be ruled out since its substitution rate is among the slowest of the gene family studied by Rocha et al. [17], only slightly faster than RecA.

**Figure 2:**
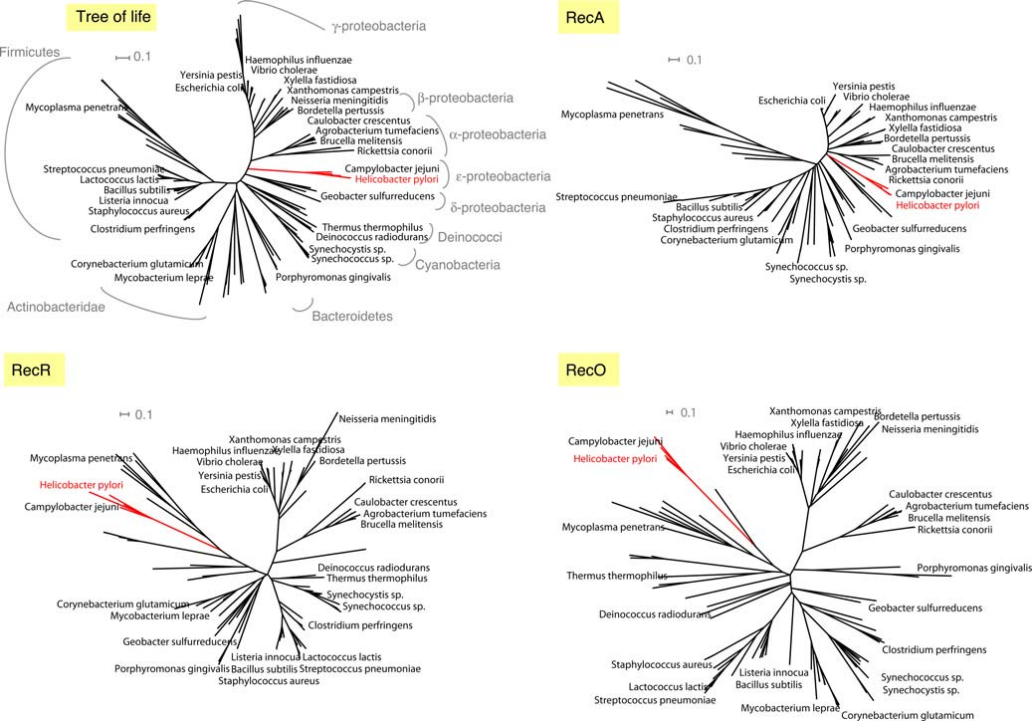
Phylogenetic trees estimated using PHYML program for RecA, RecR and RecO. The reference tree of life was derived from a concatenation of 31 orthologues occurring in 191 species [28]. The e-proteobacteria branches are in red and the H. pylori orthologues are also written as a red title.

We further explored whether the relatively high divergence observed in *H. pylori* RecO could also be found in *H. pylori* RecR. For that purpose the phylogenetic trees of both RecO and RecR were estimated using the PHYML program and compared to RecA tree and to a reference prokaryotic tree of life derived from a concatenation of 31 orthologs [28] (Figure 2). In both RecO and RecR phylogenetic trees, the e-proteobacteria do not cluster with the other proteobacteria while they do in the RecA and in the reference tree of life. Given the high evolutionary rates observed for e-proteobacteria RecR and RecO and the low bootstrap values obtained for their branches (below 70 %), biases such as long-branch attraction phenomena may explain their clustering with Firmicutes although it has no evolutionary ground [29]. As regards to e-proteobacteria, the tree analyses suggest that not only RecO but also RecR evolved significantly faster than expected with respect to other species. Such high evolutionary rate coupled with the fact that RecO is among the least conserved players of the homologous recombination pathway [17], most likely accounts for the difficulties of traditional PSI-Blast approach to detect RecO remote homologies in *H. pylori*.

### Roles of RecB, RecO and RecR in RecA-Mediated Homologous Recombination

**Figure 3:**
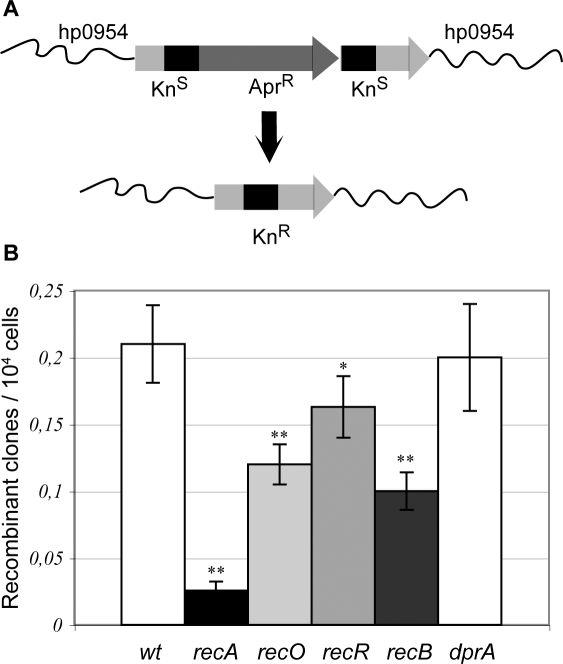
RecO, RecR and RecB participate in RecA-dependent intrachromosomal recombination. (A) Description of the experimental system. The *H. pylori* chromosome in the hp0954 region is shown in wavy lines. Arrows stand for Apr^R^ and Kn^R^ genes, and the 358-bp-long repeats of the Kn^R^ gene are represented by black blocks. Recombination between the repeats gives rise to a functional Kn^R^ gene. (B) Deletion rates are affected by mutation in *rec* genes. Recombinant rates were measured as described in *Experimental procedures*. Average and standard deviation of 11 to 32 independent determinations (see Table 1) are shown.

To determine whether the putative HR mediator orthologues actually participate in RecA-dependent recombination, we introduced into the bacterial chromosome an apramycin-resistance cassette flanked by 358 bp direct repeats within a kanamycin resistance (Kn^R^) gene. Recombination between the repeats results in deletion of the Apr^R^ marker to yield a functional Kn^R^ cassette (Figure 3A). We therefore used deletion frequencies for the parental and mutant strains as a measure of recombination efficiency (Figure 3B and Table 1). In a wild type 26695 strain, the deletion frequency was (2.1+/−0.3)×10^−5^, a frequency between 2 and 10-fold higher than the one obtained with a similar construct harbouring shorter (100-bp) repeats [30]. Inactivation of *recA* resulted in a 10-fold decrease in deletion frequency, indicating that 90% of the deletion events observed in the parental strain are originated by a RecA-dependent recombination mechanism, providing a tool to study RecA-dependent intrachromosomal HR in *H. pylori.* This is in contrast with the results obtained by Aras et al. who found that direct repeats of 100 bp or less recombined through a RecA-independent pathway. An explanation for this discrepancy might lie in the length of the repeats used. In *E. coli* plasmid systems, increasing the length of the repeat beyond 100-bp mainly results in RecA-dependent deletion events [6],[31]. Inactivation of either *recO*, *recR or recB*, resulted in significantly reduced deletion frequencies (experiments were repeated at least 24 times), 57, 76 and 48% of the wild type respectively (Table 1 and Figure 3B), demonstrating that the corresponding gene products are indeed functional and participate in HR. Because we lack extra resistance markers, double mutants could not be tested in this system. These results show that intrachromosomal recombination in *H. pylori* can make use of RecA and both RecOR and RecB initiation pathways.

**Table 1:** Intrachromosomal recombination.

Strain genotype	n^a^	Recombination rate (x10^4^)	Relative value	P value
*wt*	23	0,21 (±0,03)	= 1	
*recA*	28	0,027 (±0,005)	0,1	<2 10^−6^
*recO*	29	0,12 (±0,02)	0,6	2 10^−5^
*recR*	24	0,16 (±0,02)	0,8	0,016
*recB*	32	0,10 (±0,01)	0,5	<2 10^−6^
*dprA*	11	0,20 (±0,04)	1	0,51

a number of independent determinations.

Deletion rates were determined using the substrate described in Figure 3A.

### RecO, RecR, and RecB Roles in Recombinational Repair

**Figure 4:**
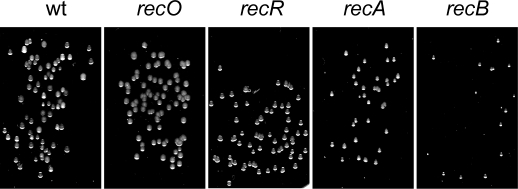
*recA* and *recB* mutants show growth impairment. For each mutant strain, appropriate dilutions of exponential growth were spread on BAB medium and incubated during 4 days.

In order to explore possible roles for the newly identified *recO* and the *recB* distant orthologues during *H. pylori* recombinational repair, we analyzed the phenotypes of simple, double or triple *rec* mutants (Table S1). Colony growth was clearly impaired in all strains defective in *recA* or *recB*, while single *recR* or *recO* mutants had no obvious growth phenotype (Figure 4). The *recA* phenotype underscores the importance of HR for normal growth. From the *recB* phenotype we conclude that a RecB-dependent pathway, that cannot be replaced by an alternative one, also plays an important role in growth. The role of RecO, RecB and RecR in recombinational repair was confirmed by the sensitivity of the different mutants to metronidazole (mtz) (Table 2). The antimicrobial mtz is known to induce DNA damage requiring recombination for its repair [13],[14],[16]. All the single mutants tested were sensitive to mtz (Table 1) with *recA* showing the highest lethality, suggesting a participation of all the corresponding proteins, including the newly identified RecO, in HR. Moreover, double mutants combining *recB* with either *recO* or *recR* were as sensitive to mtz as the *recA* mutant.

**Table 2:** Susceptibility to Metronidazol of wild-type and *rec* mutant strains.

Strain	MIC (µg/ml)
wt	0.25
*recA*	<0.016
*recB*	0.032
*recO*	0.064
*recR*	0.064
*recBrecO*	<0.016
*recBrecR*	<0.016

**Figure 5:**
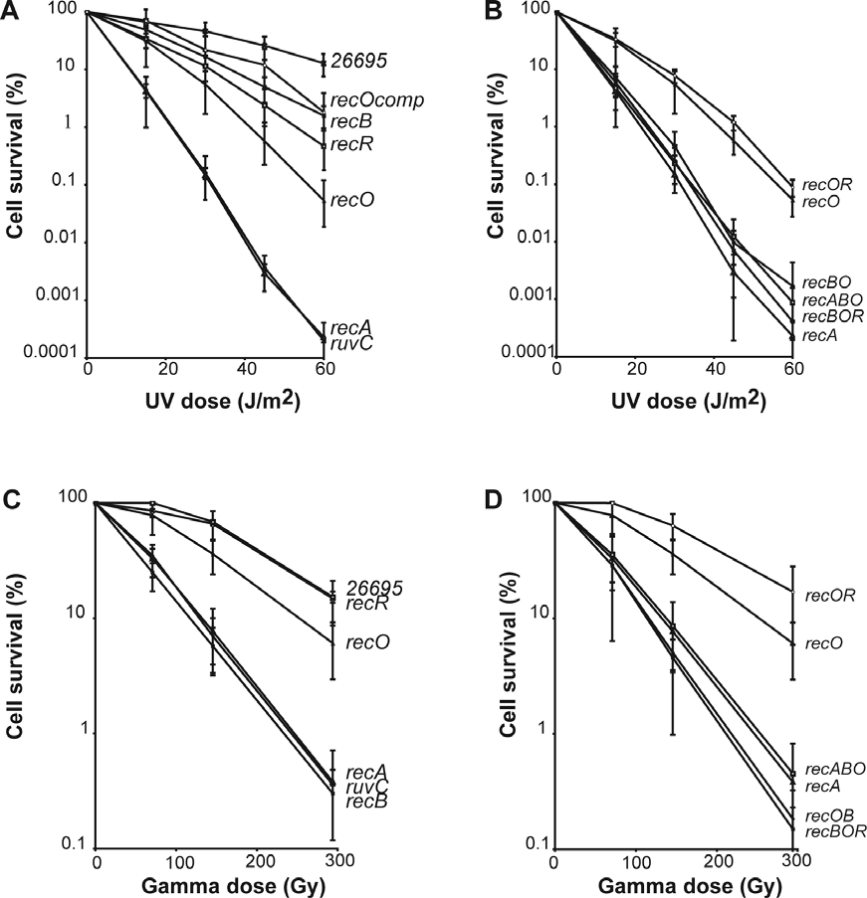
UV and gamma irradiation sensitivities of *H. pylori* mutant strains. Irradiations were performed as described in the *Materials and Methods*. Average from three to five experiments are shown. (A, B) Ultraviolet light sensitivity. (C, D) Ionizing irradiation sensitivity. *recOcomp* refers to *the recO* mutant strain expressing the HpRecO under the control of *the ureA* promoter.

As expected, *recA* mutants were very sensitive to UV (Figure 5A). Surprisingly, a *ruvC* mutant displayed exactly the same sensitivity as the *recA* strain, showing that, unlike in *E. coli*, there are no backup systems for RuvABC in the Holliday junction resolution steps. Inactivation of either *recO* or *recR* resulted in a marked sensitivity to UV, although not as pronounced as that of a *recA* mutant (Figure 5A), confirming a role of the newly identified RecO in recombinational repair. The expression of RecO from the *ureA* promoter partially restored UV resistance in a *recO* mutant, ruling out polar effects as a consequence of the mutant construction. *recB* strains displayed a UV sensitivity less marked than that of *recR* or *recO* mutants. A double *recOR* mutant had the same sensitivity than the simple *recO* mutant (Figure 5B), suggesting they belong to the same epistatic group. Double or triple mutants combining *recB* with either *recO* or/and *recR* were more sensitive to UV than single mutants and the difference of sensitivities of these double/triple mutants and *recA* strain was within experimental error, suggesting the absence of mediator activities for recombinational repair other than those involving RecB or RecOR.

In the case of ionizing radiation (IR), again *recA* and *ruvC* mutants displayed the same high sensitivity (Figure 5C). RecB-defective strains were as sensitive as *recA* or *ruvC* mutants. Resistance was recovered by expression of RecB from the *ureA* promoter in *recB* strains (data not shown). In contrast, a *recR* mutant showed no detectable change in sensitivity to IR when compared to the wild-type. The *recO* simple mutants displayed a very modest, but significant, sensitivity. The survival curves for double and triple mutants (Figure 5D), show that inactivating simultaneously *recO* and *recR* did not affect IR sensitivity and that deficiencies in either RecA, RecO or RecR did not modify the sensitivity of a *recB* strain, indicating that all the recombinational repair of IR-induced damage is mediated by the RecB-dependent pathway.

### DNA Integration into the Chromosome Following Transformation Is RecB- and RecOR-Independent

Natural transformation in *H. pylori* involves uptake of DNA from the medium followed by its incorporation into the chromosome by HR. We examined the role of the RecOR and RecB-dependent pathways in chromosomal DNA transformation. Cells from a streptomycin-resistant (Str^R^) 26695 mutant were used as a source of transforming DNA. The yield of Str^R^ colonies for the various mutant strains was then determined (Table 3). The lack of transformants in a *recA* strain confirmed the absolute requirement for RecA in transformation. Inactivating the RecOR pathway by either single or double mutations did not affect the recombination frequency. Surprisingly, RecB seemed to act as a suppressor of recombination during transformation since, inactivating *recB* not only failed to reduce transformation but actually consistently resulted in frequencies 10-fold higher than that of the parental strain, even when combined with *recO* and/or *recR* mutations. When chromosomal DNA carrying a kanamycin-resistance cassette was used, although we could not observe a significant increase in integration frequencies in *recB* strains, inactivation of the initiation pathways did not reduce the transformation frequencies (Table 3, lower part). Taken together, these results show that neither RecOR nor RecB mediate the loading of RecA during chromosomal integration of exogenous DNA, suggesting the existence of a specialized pathway facilitating RecA nucleation during transformation.

**Table 3:** Transformation frequencies in recombination mutants.

	Streptomycin resistance integration (genomic DNA)			
Strain genotype	n^a^	Recombinant frequency (×10^4^)	Relative value	P value
*wt*	20	0,29 (±0,26)	= 1	
*recA*	20	<0.0001	nd	
*recO*	10	0.51 (±0.37)	1.8	8.2 10^−2^
*recR*	8	0.25 (±0.20)	0.9	9.4 10^−1^
*recB*	8	3.26 (±1.28)	11	6.0 10^−7^
*recBrecR*	14	2.86 (±2.62)	9.9	2.8 10^−7^
*recBrecO*	6	2.62 (±3.38)	9.0	1.6 10^−2^
*recBrecRrecO*	10	4.81 (±3.66)	16.6	1.9 10^−5^
*dprA*	10	<0.0001	nd	

	kanamycin resistance integration (genomic DNA)			
Strain genotype	n^a^	Recombinant frequency (×10^4^)	Relative value	P value
*wt*	6	2.4 (±0. 4)	= 1	
*recA*	6	<0.0001	nd	
*recO*	6	4.2 (±1.5)	1.8	4.1 10^−2^
*recR*	6	1.7 (±0.4)	0.7	8.7 10^−3^
*recB*	6	1.8 (±1.0)	0.8	1.8 10^−1^
*recBrecR*	7	1.5 (±0.6)	0.6	2.6 10^−2^
*recBrecO*	6	4.9 (±1.0)	2.1	2.2 10^−3^

a number of independent determinations.

## Discussion

In this study, we unveiled the presence in *H. pylori* of a functional remote orthologue of RecO. Based on this finding, we identified RecO orthologues in all those species where standard sequence comparison techniques had identified a RecR but failed to detect a RecO. These results imply that, consistently with the biochemical and genetic studies in *E. coli*, *B. subtilis* and now *H. pylori*, the two proteins depend on each other for their activity, reflecting most likely the formation of a functional RecOR complex. We could not however identify by our procedure a proper homologue of RecF in *H. pylori*. Because this gene exhibits substitution rates almost as high as RecO, we cannot exclude that such remote homologue actually exists. Although the HpRecO shares less than 15% sequence identity with most of the previously identified orthologues, modeling predicts a structure with striking similarities to the structure of the *D. radiodurans* homologue (Figure 1A). Some differences are however worth noticing, in particular that concerning the surface charge distribution, which is clearly positive for most of the DrRecO and EcRecO surface but only locally so for the *H. pylori* orthologue. A large acidic patch can be observed on one side of the molecule and only a fraction of the OB-fold maintains the basic character (Figure 1C). Interestingly, mutation of two positively charged residues on this face of the OB-fold (shown as pink stars in Figure 1) was found detrimental for DrRecO DNA binding. In contrast, the positions of two basic residues that were found unimportant for the interaction with DNA in *D. radiodurans* (shown as yellow stars in Figure 1) are located in a region whose electrostatic potential is largely acidic in *H. pylori*. This makes of the *H. pylori* protein an interesting model to study the RecO interactions with DNA.

On the other hand, the high degree of conservation of the RecR proteins among prokaryotes was not found respected in e-proteobacteria. The phylogenetic tree analysis of RecR revealed that, as for RecO, e-proteobacteria homologues were subjected to higher evolutionary rate than expected from other proteobacteria. It raises the interesting possibility that RecO and RecR coevolved at high rate and motivate a further in-depth comparative analysis of the RecOR complex in *H. pylori*.

Our results also allow a first description of the recombination mechanisms active in *H. pylori*. Using a novel assay for RecA-dependent intrachromosomal recombination (Figure 3A), we show that the two initiation pathways, RecOR- and RecB-dependent, participate in such mechanism. The lack of additional resistance markers did not allow to rule out the possibility, although unlike, of the involvement of a third, RecOR- and RecB-independent, initiation mechanism for RecA-mediated intrachromosomal recombination. Neither could we explore the degree of overlap of the two systems. The answers to these points will need the development of new tools allowing the introduction of more than three selective markers in *H. pylori.*

The two initiation pathways participate in recombinational repair and the phenotypes of strains disabled for both of them exclude the existence of a third one. The RecOR-dependent pathway, possibly lacking a RecF component, confers resistance to UV radiation, reflecting a role in gap repair or replication fork restart. The RecB-dependent pathway is essential for repair of IR-induced lesions, most likely double strands breaks. Interestingly, these two pathways show little capacity to act as backups of each other after IR treatment, unlike the *E. coli* situation in which the RecFOR pathway can substitute for RecBCD in the repair of double strand breaks under certain conditions. In particular, the absence of a RecQ homologue in *H. pylori* could explain the inability of the RecOR pathway to process double strand breaks in a *recB* mutant. The lack of redundancy, consistent with the modest size of its genome, seems to be a common theme in *H. pylori* metabolisms. As was the case for base excision repair [32], this can greatly facilitate the study of the essential components of the homologous recombination machinery. The resolution step of *H. pylori* reflects the lack of the redundancy found in other species. Indeed, the sensitivity of *ruvC* mutants to genotoxic treatments being equivalent to that of a *recA* strain, it can be inferred that no backup system for RuvABC-dependent resolution is present in this pathogen. In *E. coli*, RecG helicase can partially substitute for RuvABC activities [33]. Although present in *H. pylori* (HP1523), HpRecG does not contribute to DNA repair [34], suggesting a different function for this putative helicase.

As in most bacterial species studied, RecA is essential for *H. pylori* recombination processes. Intriguingly, *H. pylori recA* strains are between two and three orders of magnitude more resistant to IR and UV than *E. coli recBC* or *recA* strains [20],[35]. Although this could be partially due to the lack of an SOS system in *H. pylori*, we do not understand at this point the causes of such differences.

We finally explored the requirement for recombination genes in the process of transformation. HR mediates the integration of transforming DNA into the recipient chromosome. Our results show that the mediator activities participating in recombinational repair and intrachromosomal recombination are not required for transformation. This is in contrast with *B. subtilis* transformation where, although inactivation of either AddAB (RecBC) or RecFOR pathways has a modest effect on chromosomal transformation capacity (approximately 50% reduction), double mutants deficient in both epistasis groups have transformation frequencies more than 10-fold lower than those of wild type strains [10],^36^. While in *S. pneumoniae*, RexAB (functional homologue of RecBCD*)* inactivation did not affect significantly chromosomal transformation [37], no data is available on mutants defective in the other initiation pathway. Surprisingly, our data shows that *H. pylori recB* mutants are hypertransformable, at least for some DNA substrates (Table 3). This observation puts HpRecB, together with MutS2 [38], UvrD [39] and RecG [34] in the growing list of transformation suppressors found in this pathogen. The dispensability of RecOR and RecB for transformation suggests the possibility of an alternative activity able to load RecA during transformation. An obvious candidate is suggested by a recent report showing that *in vitro* the single stranded-DNA binding protein DprA from *B. subtilis* and *S. pneumoniae* can act as a mediator in RecA filament formation on SSB-covered DNA [40]. In *H. pylori*, *dprA* mutants are completely deficient in chromosomal transformation [41]. Inactivation of *dprA* does not contribute to UV nor IR sensitivity even in strains disabled for both RecB and RecOR (Figure S3). Neither is a *dprA* mutant affected in intrachromosomal recombination (Figure 3B). These results suggest that DprA could provide a mediator activity exclusively devoted for transforming DNA. The DprA requirement for transformation underlines the role of single stranded DNA intermediates. However, the sensitivity of transformation frequencies to restriction modification [38] suggests that at some point the incoming DNA is present in the cell as a duplex. Hence, *H. pylori* represents a unique model for the study of chromosomal transformation mechanisms, specially considering their importance in horizontal gene transfer and therefore adaptation capacity and antibiotic resistance acquisition.

## Materials and Methods

### Bioinformatic Detection and Analysis of RecO

Five iterations of PSI-BLAST were performed to reach convergence on the nr_bac70f database using RecO from *Escherichia coli* as query sequence (swissprot : RECO_ECOLI) (Toolkit server [42]). About 320 homologous sequences could be retrieved but no sequence from any e-proteobacteria. At an e-value of 1.5, a sequence from *Campylobacter fetus* caught our attention because of its evolutionary proximity to *Helicobacter pylori*. Using the latter as a seed, we could retrieve homologous sequences from *H. pylori* with high confidence. The alignment made of only e-proteobacteria sequences was compared with the other RecO homologues sequences using the HHalign profile-profile comparison method and yield high probability that these sequences were evolutionary related (probability 96.2 % with e-value of 5.10^−4^) [24]. A structure of RecO in *D. radiodurans* (PDB : 1u5k) was used as template to build a model of the *H. pylori* RecO. The whole set of RecO homologues, including that of ee-proteobacteria was gathered and aligned using the Kalign algorithm [43]. This alignment is built without the knowledge of the three dimensional structure and it is known that below 20% identity alignment errors are frequent. In most variable regions the alignment was thus further optimized manually to respect that the buried positions are kept hydrophobic and that the secondary structures are the least broken by insertions. Although *H. pylori* and *D. radiodurans* RecO share only 14 % identity, a structural model of the *H. pylori* RecO could be generated. The model generated the acceptable Prosa2003 and Verify3D evaluation methods' scores (-0.59 and 0.31, respectively) with only 7 residues having a negative Verify3D score in the (50–55) loop of the protein. Evolutionary rates were calculated using the conservation scores for each amino acid position in the multiple sequence alignment were computed using the rate4site program [44]. The Bayesian method was applied for the calculation of the conservation scores using the Jones-Taylor-Thornton amino acid substitution model [45]. The conservation scores computed by rate4site were rescaled between 0 and 99 and were then mapped onto the protein by replacing the B factors in the PDB file. The most conserved residues correspond to the highest rescaled scores. Electrostatic potential analysis was performed using the APBS program. Structures were drawn with Pymol (DeLano, W.L. The PyMOL Molecular Graphics System (2002) http://www.pymol.org) and multiple sequence alignment with JalView [46].

### Phylogenetic Analyses of RecA, RecR, and RecO

Multiple sequence alignments gathering a maximum number of prokaryotic sequences were built for RecA and RecR. Homologues were retrieved after one iteration of PSI-Blast on the nr database and their full sequences were realigned using the Kalign algorithm [43]. As regards RecO, the alignment resulting from the profile-profile comparison further optimized by hand (see above) was used. After filtering for redundancy about 500 sequences were considered in the multiple sequence alignments of each protein. We derived Maximum Likelihood (ML) phylogenetic inferences using PHYML [47] and applying the JTT matrix. Our model of sequence evolution assumed that there were two classes of sites, one class being invariable and the other class being free to change. The rate variation across these sites was assumed to follow a gamma shape distribution calculated using a discrete approximation with four categories of sites. Support for the hypotheses of relationships were estimated using 10 bootstrap replicates. The resulting trees were pruned and represented using the Dendroscope program to restrict the analysis to a subset of about 100 representative bacteria [48]. The reference tree of life was obtained from the iTOL server [28],[49].

### *H. pylori* Strains and Growth Conditions

All *H. pylori* strains used were in the 26695 background [23] and are listed in Table S1. Plate cultures were grown at 37°C under microaerobic conditions on blood agar base medium supplemented with an antibiotic mix and 10% defibrillated horse blood (BAB). Plates were incubated from 24 h up to 5 days depending on the experiment or the mutant selected.

To generate the corresponding mutant derivatives, the gene of interest cloned into pILL570 was disrupted, leaving the 5′ and 3′ ends (300 bp) of the gene, by a cassette carrying either a non-polar kanamycin- (Kn) [50], an apramycin- (Apr) or a Chloramphenicol- (Cm) [51] resistance genes.

DNA was introduced into *H. pylori* strains by natural transformation and selection after 3 to 5 days of growth on either 20 µg/ml Kn, 12,5 µg/ml Apr or 8 µg/ml Cm. Allelic replacement was verified by PCR. Double or triple mutant strains were obtained by plasmid or genomic DNA transformation of single mutant or by mixing two mutant strains together before plating the mix on double or triple selection. A minimum of two mutants obtained independently for each construction (excepted for *recArecOrecR*) were studied for each phenotype.

For complementation of *recO* or *recB* mutants the corresponding open reading frames were cloned into pADC downstream of the *ureA* promoter [41] and introduced into the mutant strains as describe above, using Cm^R^ as selection for integration of the construct at the *ure* operon. The protine of interest is then expressed using the *urea* strong promoter.

### Sensitivity Assays

For UV sensitivity assays, bacterial cell suspensions were serially diluted and 10 µl of each dilution was spotted on BAB plates. Cells were irradiated with 0, 15, 30, 45, and 60 J of 264-nm UV light delivering 1 J/m2/s. Gamma irradiation was performed using a ^137^Cs source delivering 30 Gy/min. Survival was determined as the number of cells forming colonies on plates after a given irradiation divided by the number of colonies from non-irradiated cells. Susceptibility to metronidazole was assessed by the Epsilometer test (E-test; AB Biodisk, Solna, Sweden).

### Construction of the Intragenomic Recombination Substrate

To construct the Kn_du_::Apr recombination substrate (Figure 3A), first, pTZApra, a derivative of pTZ19U, was constructed by cloning the Apr gene responsible for apramycin resistance into pTZ19U between *Kpn*I and *Bam*HI sites. From pILL570-rdxA::Kn (the plasmid harbouring *rdxA* gene disrupted by Kn cassette), we amplified with *Dra*III and *Nae*I restriction sites the *rdxA* 5′ extremity (318 bp) with the first part of Kn gene (555 pb). We cloned this fragment into pTZApra between *Dra*III and *Nae*I sites and obtained plasmid ptZApra954K5. Then, from PILL570-rdxA::Kn again, we amplified, with *Xba*I and *Hind*III restriction sites, the downstream part of Kn gene (604 pb) with *rdxA* 3′ extremity (361 bp) and cloned this fragment into ptZApra954K5 at the corresponding site. A *Nae*I/*Kpn*I fragment (301 bp) was removed to avoid recombination between a small direct repeat, obtaining plasmid pTZ954-Kn_du_-Apra. This results in an Apr^R^ cassette flanked by 358 bp direct repeats of the Kn^R^ gene (Figure 3A).

The Kn_du_::Apra structure was then inserted into strain LR360 by replacement of the intact Kn gene present in *rdxA* and selection on apramycin. Integration of the recombination substrate in strains LR436 and LR437 was verified by PCR. Mutants in the *rec* genes were obtained by transformation of these two strains with the corresponding plasmid as described above.

### Deletion Assay

Strains to be tested were grown on BAB plates containing apramycin (12,5 µg/ml). When they reached the exponential step (24 h), 25 µl of resuspended cells (2,5 10^5^ cells) were spotted on BAB plates. After 24 hours at 37°C, appropriate dilutions were plated on BAB with and without 20 µg/ml Kn and incubated for 3 to 5 days. The recombination rates and their standard deviations were calculated from 11 to 32 independent experiments by the method of the median [52].

### Natural Transformation Assay

200 ng of DNA (genomic of either strain LR133 (Str^R^) or LR360 (Kn^R^) was mixed with 15 µl of resuspended exponentially growing cells (2,5 10^5^ cells). Mixes were spotted on BAB plates. After 24 hours at 37°C, dilutions of the resuspended spots were plated on BAB with and without the appropriate antibiotic (50 µg/ml Str or 20 µg/ml Kn) and incubated for 3 to 5 days. Transformation frequency was calculated as the number of resistant colonies per recipient cfu. P values were calculated using the Mann-Whitney U test.

## Supporting Information

Figure S1 Multiple sequence alignment of RecO homologues. Secondary structure elements observed in the structure of DrRecO are shown on top. Grey bars above the alignment indicate the positions in contact with RecR in the Xray structure of the DrRecOR complex. Stars indicate the residues that were mutated in DrRecO and whose binding properties to RecR and DNA were characterized. Pink stars indicate the position of the two mutations that disrupted RecO DNA binding properties while yellow stars indicate the positions of two mutations that only partially affected DrRecO DNA binding [26].
(12.59 MB TIF)

Figure S2 Electrostatic properties of RecO homologues. Ribbon representation of the DrRecO Xray structure and of HpRecO and EcRecO models (B) Representation of the electrostatic potential calculated with the APBS program and projected on the molecular surface of DrRecO, HpRecO and EcRecO, shown under two opposite orientations. Pink stars indicate the position of the two mutations that disrupted RecO DNA binding properties while yellow stars indicate the positions of two mutations that only partially affected DrRecO DNA binding [26].
(7.16 MB TIF)

Figure S3 DprA-defective strains are not affected in recombinational repair. Survival of dprA mutants to UV (A) or gamma radiation (B) increasing doses.
(4.18 MB TIF)

Table S1 Strains.
(0.09 MB DOC)
