# Supplementary figures and images for: Unveiling Novel RecO Distant Orthologues Involved in Homologous Recombination
Source: PLoS Genet. 2008 Aug 1;4(8):e1000146. doi: 10.1371/journal.pgen.1000146 (PMC2475510; doi:10.1371/journal.pgen.1000146)

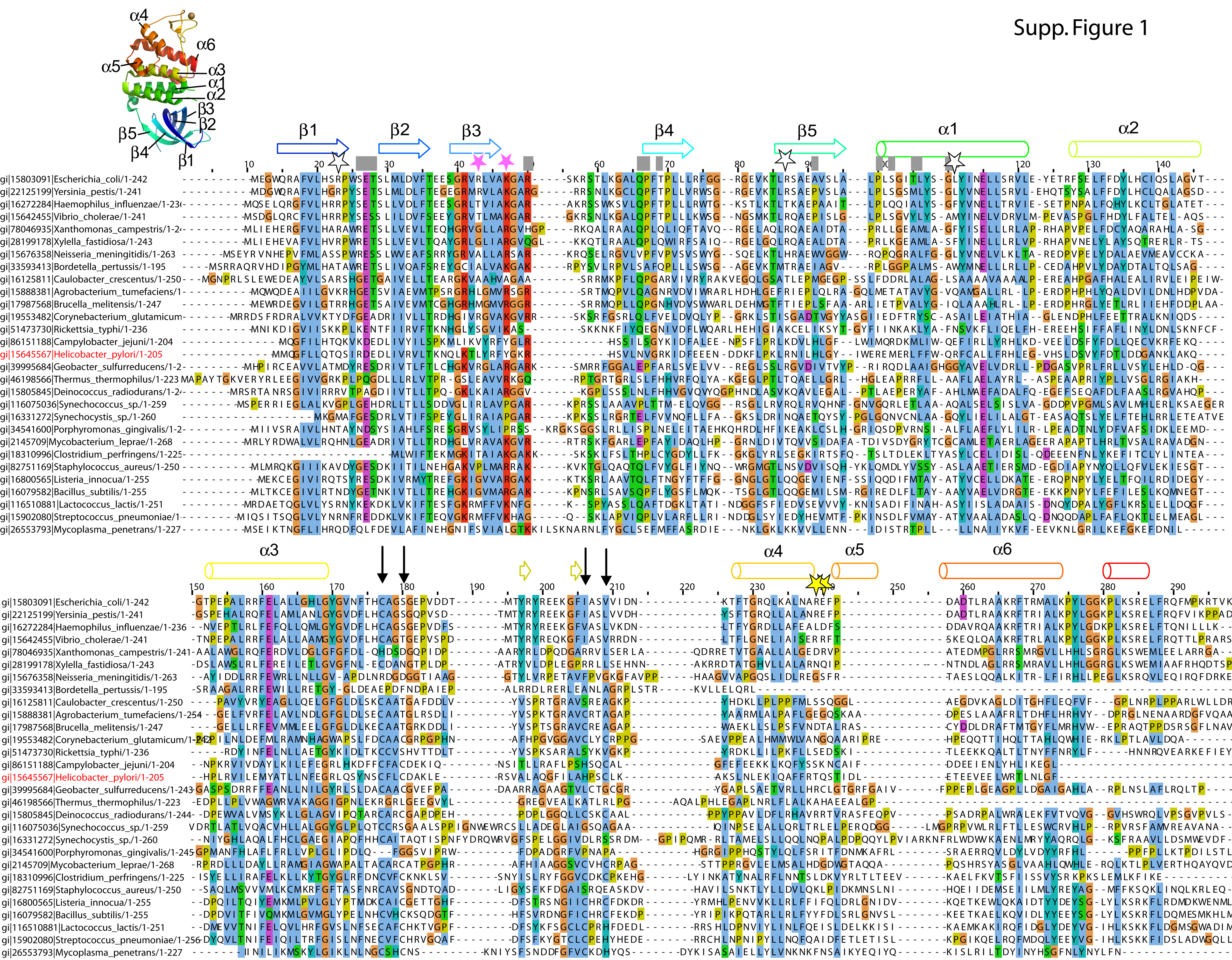

Supplement: Figure S1 — Multiple sequence alignment of RecO homologues. Secondary structure elements observed in the structure of DrRecO are shown on top. Grey bars above the alignment indicate the positions in contact with RecR in the Xray structure of the DrRecOR complex. Stars indicate the residues that were mutated in DrRecO and whose binding properties to RecR and DNA were characterized. Pink stars indicate the position of the two mutations that disrupted RecO DNA binding properties while yellow stars indicate the positions of two mutations that only partially affected DrRecO DNA binding [26]. (12.59 MB TIF) [file pgen.1000146.s001.tif]

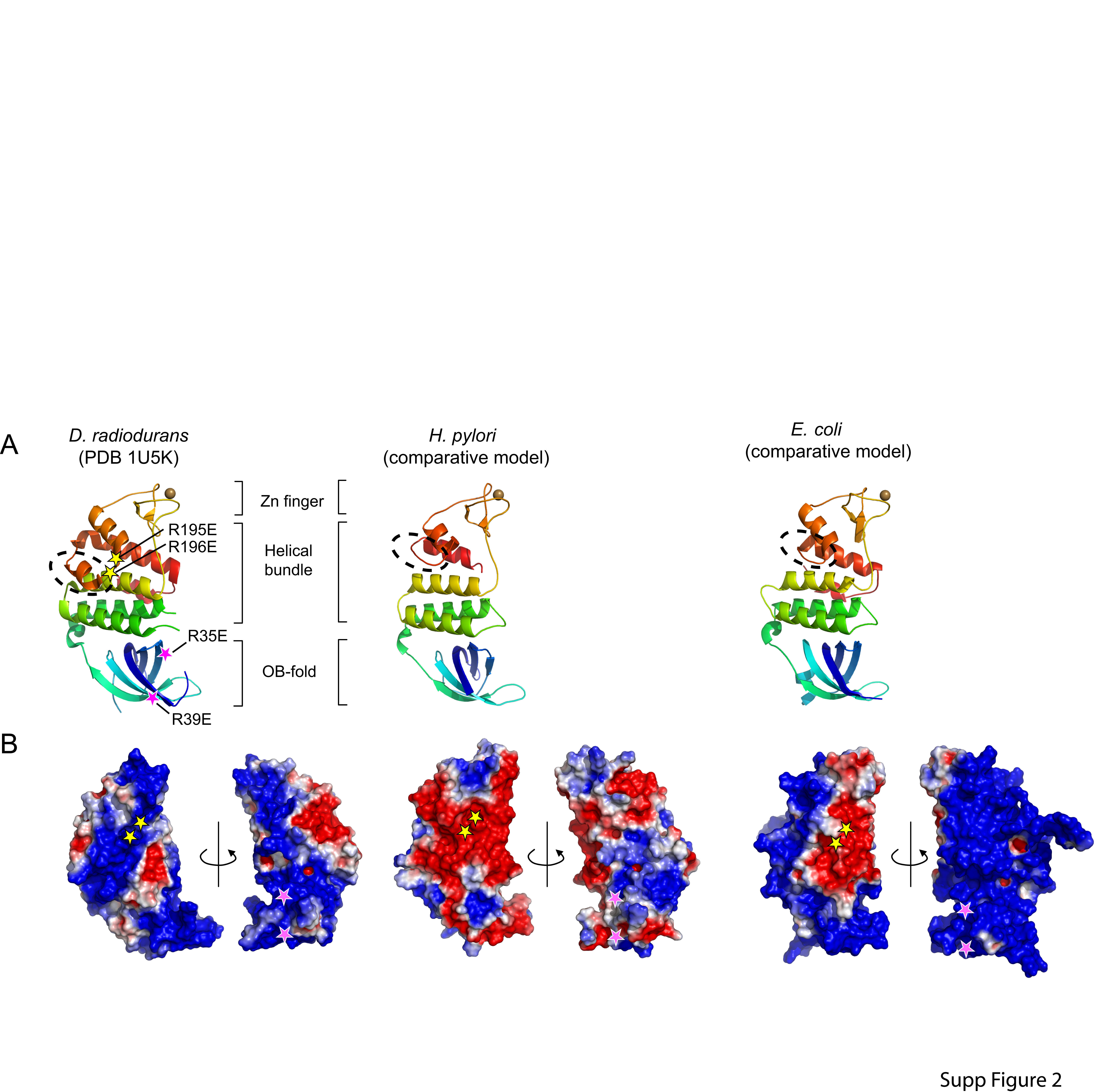

Supplement: Figure S2 — Electrostatic properties of RecO homologues. Ribbon representation of the DrRecO Xray structure and of HpRecO and EcRecO models (B) Representation of the electrostatic potential calculated with the APBS program and projected on the molecular surface of DrRecO, HpRecO and EcRecO, shown under two opposite orientations. Pink stars indicate the position of the two mutations that disrupted RecO DNA binding properties while yellow stars indicate the positions of two mutations that only partially affected DrRecO DNA binding [26]. (7.16 MB TIF) [file pgen.1000146.s002.tif]

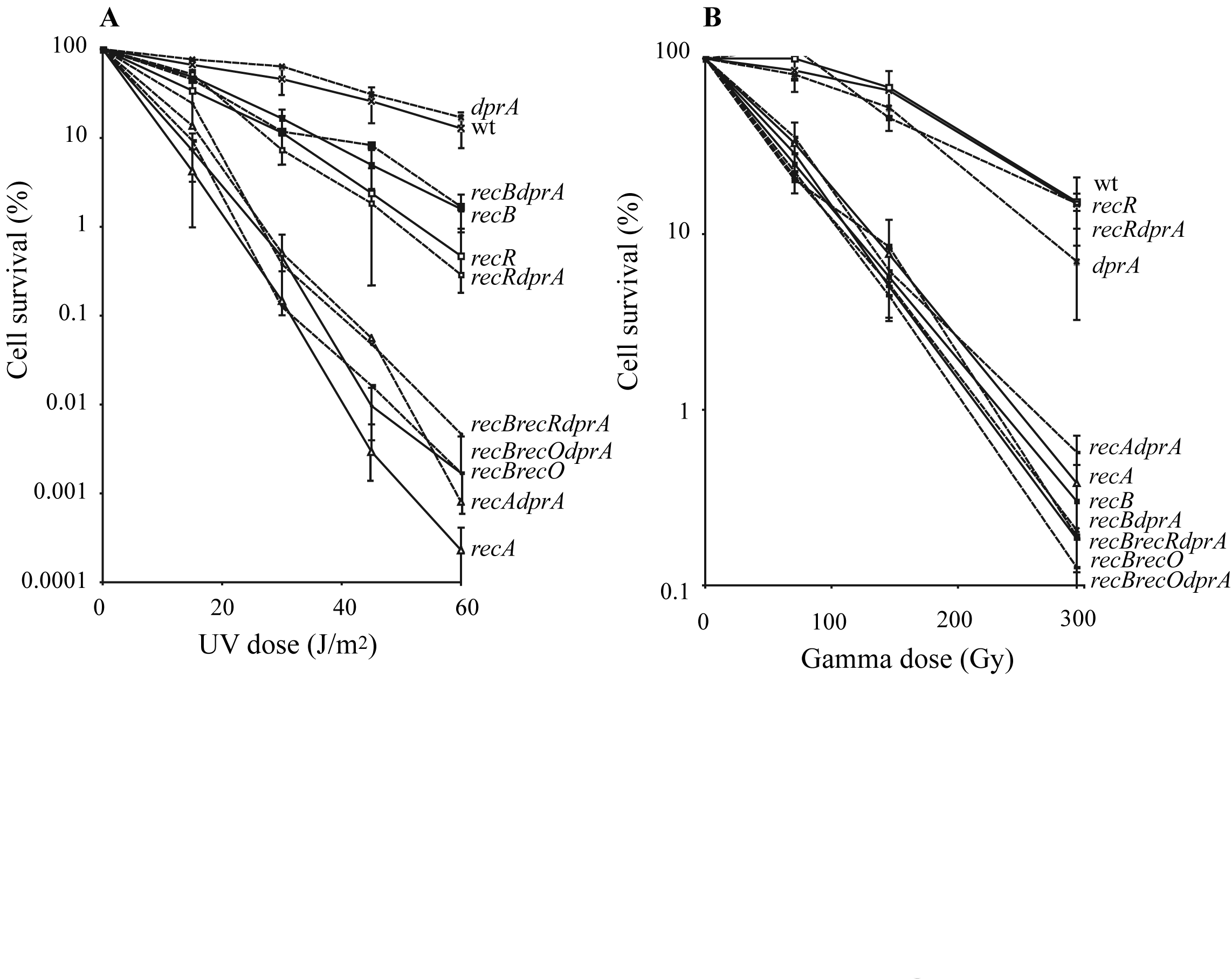

Supplement: Figure S3 — DprA-defective strains are not affected in recombinational repair. Survival of dprA mutants to UV (A) or gamma radiation (B) increasing doses. (4.18 MB TIF) [file pgen.1000146.s003.tif]
