# Supplementary material for: Unveiling Novel RecO Distant Orthologues Involved in Homologous Recombination
Source: PLoS Genet. 2008 Aug 1;4(8):e1000146. doi: 10.1371/journal.pgen.1000146 (PMC2475510; doi:10.1371/journal.pgen.1000146)
Supplement: Table S1 — Strains. (0.09 MB DOC) [file pgen.1000146.s004.doc]

**Table S1A.** Correspondence between HP open reading frame numbers and rec genes:

| Rec gene | *recA* | *recB* | *recO* | *recR* | *dprA* | *ruvC* | *rdxA* |
| --- | --- | --- | --- | --- | --- | --- | --- |
| Orf number | hp0153 | hp1553 | hp0951 | hp0925 | hp0333 | hp0877 | hp0954 |

**Table S1B.** *H. pylori* mutant strains

| Strain 26695 | Relevant genotype |
| --- | --- |
| LR 1 | *rec+* |
| LR116/LR117 | *ruvC::Kn* |
| LR121 | *recA::Kn* |
| LR293/LR294 | *recA::Cm* |
| LR312/LR313 | *recA::Apra* |
| LR303/LR304 | *recB::Cm* |
| LR320/LR322 | *recB::Apra* |
| LR394/LR395 | *recO::Kn* |
| LR420/LR421 | *recO::Cm* |
| LR122/LR123 | *recR::Kn* |
| LR291/LR292 | *recR::Cm* |
| LR412/LR413 | *dprA::Kn* |
| LR360 | *rdxA::Kn* |
| LR309/LR310 | *recB::Cm recA::Apra* |
| LR418/LR419 | *recA::Apra recO::Kn* |
| LR306/LR307 | *recR::Cm recA::Apra* |
| LR330/LR331 | *recB::Apra recR::Cm* |
| LR334/LR335 | *recB::Cm recR::Kn* |
| LR350/LR351 | *recB::Apra recO::Cm* |
| LR414/LR415 | *recR::Cm recO::Kn* |
| LR429/LR430 | *recR::Kn recO::Cm* |
| LR356/LR357 | *recB::Apra recR::Cm recA::Kn* |
| LR358/LR359 | *recB::Cm recR::Kn recA::Apra* |
| LR377 | *recB::Apra recO::Cm recA::Kn* |
| LR416/LR424 | *recR::Cm recA::Apra recO::Kn* |
| LR364/LR365 | *recB::Apra recO::Cm recR::Kn* |
| LR417/LR422* | *recA::Apra dprA::Kn* |
| LR398/LR399 | *recR::Cm dprA::Kn* |
| LR400/LR401 | *recB::Cm dprA::Kn* |
| LR402/LR403 | *recB::Apra recR::Cm dprA::Kn* |
| LR406/LR407 | *recB::Apra recO::Cm dprA::Kn* |
| LR133 | *str^R^* |
| LR360 | *rdxA::Kn* |
| LR408 | LR394(*recO::Kn)*  pADC recO Cm |
| LR410 | LR395(*recO::Kn)*  pADC recO Cm |
| LR453/LR426 | LR322(*recB::Apra)*  pADC recB Cm |
| LR436/LR437 | *rdxA::Kn_du_::Apra* |
| LR438/LR439 | LR436 (*rdxA::Kn_du_::Apra) recA::Cm* |
| LR440/LR441 | LR437 (*rdxA::Kn_du_::Apra) recA::Cm* |
| LR449/LR450 | LR436 (*rdxA::Kn_du_::Apra) recB::Cm* |
| LR451/LR452 | LR437 (*rdxA::Kn_du_::Apra) recB::Cm* |
| LR444/LR445 | LR436 (*rdxA::Kn_du_::Apra) recO::Cm* |
| LR442/LR443 | LR437 (*rdxA::Kn_du_::Apra) recO::Cm* |
| LR446/LR447 | LR436 (*rdxA::Kn_du_::Apra) recR::Cm* |
| LR448 | LR437 (*rdxA::Kn_du_::Apra) recR::Cm* |
| LR460/LR461 | LR436 (*rdxA::Kn_du_::Apra) dprA::Cm* |
| LR462/LR463 | LR437 (*rdxA::Kn_du_::Apra) dprA::Cm* |
